# Serum magnesium and high-sensitivity C-reactive protein as a predictor for gestational diabetes mellitus in Sudanese pregnant women

**DOI:** 10.1186/s12884-019-2429-x

**Published:** 2019-08-16

**Authors:** Warda Naser, Ishag Adam, Duria A. Rayis, Mohammed A. Ahmed, Hamdan Z. Hamdan

**Affiliations:** 1 Faculty of Medicine, Al-Neelain University, P.O Box 12702, 11111 Khartoum, Sudan; 2 Faculty of Medicine, University of Khartoum, P.O Box 102, 11111 Khartoum, Sudan; 3 Al-Neelain Institute for Medical Research (NIMR), Al-Neelain University, Khartoum, Sudan

**Keywords:** Magnesium, CRP, Pregnancy, Insulin resistance, Glucose, Sudan

## Abstract

**Background:**

Gestational diabetes mellitus (GDM) is a big health problem that adversely affects both the maternal and perinatal outcomes. We aimed to predict the development of GDM in the first trimester using high sensitivity C-reactive protein (hs-CRP) and serum magnesium.

**Methods:**

The study conducted in the antenatal care clinic of Saad Abualila Hospital (Khartoum, Sudan). Pregnant women were enrolled in this longitudinal cohort study during first trimester ≤14 weeks of gestation. Serum hs-CRP and magnesium concentrations were measured between weeks 11 and 14 of gestation. Glucose tolerance test and fasting plasma insulin (FPI) measurement were performed between 24 and 28 weeks gestational age. To assess insulin sensitivity and β-cell function, Homeostatic Model Assessment Insulin Resistance (HOMA-IR), HOMA-β indices and Quantitative Insulin Sensitivity Check Index (QUICKI) were calculated and used.

**Results:**

Out of the 126 who completed the study 19 (15%) were diagnosed as GDM. The median (interquartile) of FBG was significantly higher in women with GDM [81 (70–95) vs. 67(60–75) mg/dl; *P* = < 0.001] compared to women without GDM. There was no significant difference in hs-CRP, serum magnesium, HOMA-IR, QUICKI and HOMA- β between women with GDM and women without GDM. No correlation was observed between body mass index (BMI), serum magnesium, hs-CRP, FBG and insulin levels.

**Conclusions:**

First trimester hs-CRP and serum magnesium levels were not correlated with the later development of gestational diabetes in this setting.

## Background

Gestational diabetes mellitus (GDM), according to American Diabetes Association (ADA) it is defined as a true diabetes that firstly recognized at first or second trimester and not proceeded by either type 1 or type 2 diabetes [1]. GDM among the major medical complication of pregnancy that can result in adverse health consequences for both the mother and the outcome [2]. Women with GDM are vulnerable to develop metabolic syndrome/type 2 diabetes later in life [3, 4]. Likewise, children born to mothers with GDM are threaten by glucose intolerance and obesity [5].

Screening to identify women at risk for GDM among pregnancies may allow more time for interventions and can yield a reduction in both GDM and its related adverse effects. Unfortunately, screening for GDM is commonly practiced after the 24^th^ gestational week where there is a possible delay in implementing the desired/ planned interventions e.g. pharmacological therapy, diet, and exercise [6]. Therefore, there is a need for screening methods for GDM in detecting the risk of GDM in the first trimester of the pregnancy. Metabolic studies have detected various biomarkers that may be useful in early screening for GDM [7]. Low serum magnesium accompanied with high hs-CRP has been reported as a predictor for GDM in obese pregnant women [8]. It is known that magnesium promotes blood glucose reduction as it enhances the activity of the glucose transporter- 4 [9]. Moreover, it is directly involved in signalling transduction of the insulin receptor [10]. On the other hand, high serum hs-CRP increased in response to inflammatory reactions owing to insulin resistance observed in GDM [11]. Recent findings suggest serum magnesium and C-reactive protein could be used as predictors for GDM [8, 12–19]. However, the results of these studies were variable and there is a need for further research in the different settings. Thus, the current study was conducted to investigate the correlation between serum magnesium, high sensitivity CRP (hs-CRP), glycaemic and insulin sensitivity index and to assess the performance of magnesium and hs-CRP in predicting GDM in the first trimester.

## Materials and methods

A longitudinal cohort study was conducted at the antenatal care clinic of Saad Abualila Hospital (Khartoum, Sudan) during the period of January–November 2015. All women with singleton pregnancy, started antenatal care follow-up in the first trimester (≤14 weeks of gestation) were approached to participate in the study. Women with chronic disease e.g. thyroid disease, hypertension, renal disease, diabetes, liver disease and on medication were excluded from the study. After signing an informed consent, socio-demographic and obstetric characteristics were gathered by questionnaire (age, parity, gestational age, education, occupation and history of miscarriage). The weight and height were measured and body mass index (BMI) was computed (kg/m^2^). Pregnancy and its duration was confirmed by ultrasound operated in the clinic by a senior obstetrician. Then 5 ml of blood were withdrawn in EDTA tube, 2 mLs of it was used for hemogram and the rest were centrifuged and stored at − 20 °C until the assay of hs-CRP level, which was performed by EUROIMMUN analyser I-2P (Luebeck, Germany). The same procedure was used to measure insulin level lately in the third trimester. Atomic absorption spectrophotometry (SOLAAR, Atomic Absorption Spectrophotometer, Thermo Electron, Cambridge, UK) was used to determine levels of serum magnesium.

Then the enrolled women were followed in the antennal care clinic every month. According to the hospital policy, the glucose tolerance test was performed at 24–28 weeks of gestation. Recommended blood glucose levels determined by International Association of Diabetes and Pregnancy Study Groups (IADPSG) and ADA were used to detect GDM among cases in this study. Which is described very briefly as one or more of the following blood glucose values met or exceeded; fasting blood glucose (FBG) ≥92 mg/dl, 1-h blood glucose was ≥180 mg/dl, and 2-h blood glucose ≥153 mg/dl, after 75-g oral glucose load [1, 19]. The study is adhered to STROBE guidelines [20].

Glucose oxidase test was used to assess the level of blood glucose (Shino-Test Corp. Tokyo, Japan). Insulin resistance was measured using the homeostasis model assessment (HOMA) of insulin resistance index (HOMA-IR) = fasting glucose level (mg/dl) × fasting insulin level (μU/ml) / 405 [21] and the quantitative insulin sensitivity check index (QUICKI) = 1 / [log fasting insulin level (μU/ml) + log fasting glucose level (mg/dl)] [22]. Insulin secretion was indicated by the HOMA of β-cell function (HOMA-β) (%) = 360 × fasting insulin level (μU/ml) / [fasting glucose level (mg/dl) − 63, [22].

Sample size calculated based on previous data [23], it was assumed that 13.9% of the pregnant women would experience gestational diabetes [23]. Allowing for 10% of participants to be lost to follow-up, a necessary sample size of 166 participants.

## Statistics

Statistical analysis was performed using Statistical Package for the Social Sciences (SPSS) for Windows, version 20.0 (SPSS Inc., Chicago, IL, USA). Proportions of the studied variables were expressed in numbers (%). Continuous data were checked for normality using Shapiro-Wilk test. The means (standard deviations) or median (interquartile) were used to describe the studied variables if normally and abnormally distributed, respectively. Spearman correlation was conducted between BMI, magnesium, hs-CRP, FBG and insulin sensitivity indices. Student’s *t*-test and Mann-Whitney *U* test _ if the data were not normally distributed_ were used to evaluate the mean differences between the studied groups. *P* <  0.05 was considered statistically significant.

## Result

One hundred and sixty women were initially enrolled, 126 (78.7%) women completed the follow-up, have complete data and analysed here. The main reason for the loss of follow-up was change of the address. The mean (SD) of gestational age in early pregnancy 10.2(2.4) and it was 26.5 (1.2) weeks at the time of the glucose tolerance testing.

General characteristics of the 126 enrolled women are shown in Table 1. Fifty-nine (46.8%) of the women were primiparae and 19 (15%) women had GDM.

**Table 1:** General characteristics of pregnant Sudanese women enrolled in early pregnancy

Variable	*N* = 126
The mean (SD) of	
Age, years	27.7 (5.6)
Parity	0.9 (1.3)
Body mass index, kg/m^2^	27.2(5.2)
Hemoglobin, g/dl	10.9(1.2)
Number (%) of	
Rural residence	20(16.7)
Education level ≤ secondary level	46 (13.6)
Housewives	252(74.6)
History of Miscarriage	31(25.8)

There was no correlation between, BMI, serum magnesium, hs-CRP, FBG and insulin level in the enrolled pregnant women (*n* = 126), Table 2.

**Table 2:** Spearman correlations between magnesium, hs-CRP, BMI, fasting blood glucose and insulin in the pregnant women (*n* = 126)

Variables	Magnesium		hs-CRP		Insulin		FBG	
	*r*	*P*	*r*	*P*	*r*	*P*	*r*	*P*
BMI	− 0.036	0.692	0.083	0.355	− 0.007	0.938	0.113	0.938
Magnesium			0.152	0.088	− 0.193	0.029	0.172	0.053
hs-CRP					0.133	0.136	− 0.046	0.611
Insulin							−0.065	0.668

While FBG was significantly higher in women with GDM, BMI, serum magnesium, insulin, HOMA-IR, QUICKI and HOMA-β were not different in women with GDM (*n* = 19) and women who had no GDM (*n* = 107), Table 3.

**Table 3:** Comparing median(interquartile) level of body mass index, hs–CRP, magnesium, glycaemic and insulin sensitivity indices between women with GDM and controls

Variable	Women with GDM (*n* = 19)	Women without GDM (*n* = 107)	*P*
Body mass index, kg/m^2^	27.4 (23.3–31.2)	26.6 (24.3–29.6)	0.904
Magnesium, mg/dl	1.8 (1.6–2.0)	1.7 (1.5–1.9)	0.245
hs-CRP, mg/l	1.8 (1.7–2.19)	1.82 (1.69–2.100)	0.912
Insulin, μU/ml	4.1 (2.2–12.9)	6.1 (3.2–12.5)	0.338
Fasting blood glucose, mg/dl	81.0 (70.0–95.0)	67.0 (60.0–75.5)	< 0.001
HOMA-IR	1.1 (0.34–0.46)	0.96 (0.54–2.02)	0.980
QUICKI	0.37 (0.33–0.46)	0.38 (0.34–0.42)	0.980
HOMA-β, %	93.0 (93.0–279.0)	102.6 (162.0–405.2)	0.602

## Discussion

The current study shows no significant difference between serum magnesium, FBG and insulin resistance indices among women with GDM and women without GDM. Similarly, Nabouli et al. reported no significant difference in the serum magnesium level between women with GDM and women without GDM [16]. However, a significantly lower serum magnesium level, its association with insulin sensitivity and with fasting insulin in mothers with GDM have been reported before [8, 17, 18, 24]. Moreover, magnesium supplementations for magnesium deficient GDM patients’ result in a decrease of FBG, insulin, HOMA-IR, HOMA-B, hs-CRP and an increase of QUICKI [24]. Perhaps, hypomagnesemia lead to inadequate beta-cell compensation for the decrease in insulin sensitivity [25]. Interestingly, postpartum serum magnesium level was found to be a possible predictor for type 2 diabetes mellitus development in women with GDM [26]. We have previously observed a significantly lower median level of serum magnesium in diabetic patients with diabetic retinopathy compared with diabetic without diabetic retinopathy [27].

The hs-CRP level in the current study was not statistically different between women with GDM and women without GDM. This goes with Syngelaki et al. findings [14]. In contrast Fatema et al. and Ozgu-Erdinc et al. have recently reported a good performance (sensitivity and specificity) of hs-CRP in predicting GDM [12, 13]. Moreover, hs-CRP has been reported to be associated with insulin resistance, insulin index and GDM [15]. However, many reports claimed that CRP is positively correlated with pre-pregnancy BMI [28, 29]. Perhaps, our finding is partially attributed to the no difference observed in BMI and hs-CRP (which were expected to be higher in women with GDM). Yet Rota et al. [30] reported that the serum hs-CRP levels were significantly higher in GDM women despite no difference in the pre pregnancy BMI. In the later study glucose intolerance and weight gain during pregnancy were the main factors which affect hs-CRP levels. In our study BMI, hs-CRP were taken in early pregnancy without follow-up values. This point (measurement in early pregnancy) could be another explanation of the non-significant difference in hs-CRP in our study. Inflammatory mediators secreted from adipose tissue and placenta can lead to escalating low-grade inflammation during pregnancy specially in the third trimester [31, 32]. It worth mentioning that, almost 75% of our patients have BMI < 30 and this finding is not astonishing as the prevelance of pregnant women who have BMI < 30 in Sudan is 79% [33]. Alternatively, perhaps in GDM the insulin resistance is an effect of impairment of insulin action, rather than insulin secretion (similar to type 2 DM patients) with declining of insulin receptors and impairing intracellular glucose transport [34].

## Conclusions

In summary, we fail to show any prediction of first trimester hs-CRP and serum magnesium with the development of GDM in this setting. Also, no correlation has been observed between insulin hormone level and insulin resistance indices, magnesium levels and hs-CRP.

This study has many limitations, firstly; the current study recruited small sample size and under power. The number of women with GDM is very low and comparison of them with women without GDM might not be reasonable. Secondly; other inflammatory markers e.g. cytokines were not measured and infections that might have effects on hs-CRP were not ruled out. Therefore, larger sample size with longitudinal study design is needed.

## Data Availability

The datasets used and/or analysed during the current study are available from the corresponding author on reasonable request.

## Abbreviations

ADA: American Diabetes Association
BMI: Body Mass Index
FBG: Fasting Blood Glucose
FPI: Fasting Plasma Insulin
GDM: Gestational Diabetes Mellitus
HOMA-IR: Homeostatic Model Assessment Insulin Resistance
hs-CRP: High Sensitivity C-Reactive Protein
IADPSG: International Association of Diabetes And Pregnancy Study Groups
QUICKI: Quantitative Insulin Sensitivity Check Index
SPSS: Statistical Package for the Social Sciences
